# Regression of Nonalcoholic Fatty Liver Disease Reduces the Development of Coronary Artery Calcification: A Longitudinal Cohort Study

**DOI:** 10.1016/j.gastha.2023.08.004

**Published:** 2023-08-16

**Authors:** D.H. Sinn, D. Kang, E. Guallar, S.C. Choi, J. Cho, G.-Y. Gwak

**Affiliations:** 1 Department of Medicine, Samsung Medical Center, Sungkyunkwan University School of Medicine, Seoul, South Korea; 2 Department of Clinical Research Design and Evaluation, SAIHST, Sungkyunkwan University, Seoul, South Korea; 3 Center for Clinical Epidemiology, Samsung Medical Center, Sungkyunkwan University, Seoul, South Korea; 4 Departments of Epidemiology and Medicine, and Welch Center for Prevention, Epidemiology and Clinical Research, Johns Hopkins Medical Institutions, Baltimore, Maryland; 5 Center for Health Promotion, Samsung Medical Center, Sungkyunkwan University, Seoul, South Korea

Nonalcoholic fatty liver disease (NAFLD) is characterized by fat infiltration in the liver without significant alcohol intake or other obvious causes.^1^ NAFLD is a hepatic manifestation of the metabolic syndrome, and is associated with an increased risk of cardiovascular morbidity and mortality.^1^ While NAFLD is potentially reversible,^1^ it is unclear if reversal of hepatic fat accumulation is associated with a clinical benefit, such as reduced cardiovascular risk. Coronary artery calcium (CAC) scores represent the extent of coronary atherosclerosis and independently predict future risk of cardiovascular events.^2^ In this study, we assessed whether NAFLD regression is associated with a reduced risk of developing coronary artery calcification in a large sample of adult men and women with NAFLD.

This analysis included men and women of 20 years of age or older who underwent at least annual or biennial health screening examinations including abdominal ultrasound (US) imaging at the Samsung Medical Center between 2001 and 2016. We included 10,693 participants who had fatty liver diseases in an initial exam and participated at least in 2 additional exams. The second exam was used to determine change in NAFLD status (persistence vs regression) and the baseline CAC status, and the third and any subsequent visits were used to determine CAC follow-up. Then, we excluded participants with alcohol intake ≥ 30 g/day in men or ≥ 20 g/day in women (N = 870), positive hepatitis B surface antigen or anti-hepatitis C virus antibodies (N = 397), liver cirrhosis (N = 293), a history of cancer (N = 645) or cardiovascular diseases (N = 383), use of aspirin, warfarin, or anticoagulants (N = 1731), and missing data on alcohol intake (N = 643). We further excluded participants with CAC > 0 at the second visit (N = 2647) and participants without follow-up CAC measurements (N = 1405). As we excluded participants with secondary causes for hepatic steatosis, fatty liver found on the US exam was considered as NAFLD.^3^ Since study participants could have more than one exclusion criteria, the final sample size was 2228 participants. The study was approved by the Institutional Review Board of the Samsung Medical Center.

Imaging data for the evaluation of CAC was acquired using Brilliance 40 (Philips Medical Systems), VCT LightSpeed 64 (GE Healthcare), or Discovery 750HD (GE Healthcare) multidetector computed tomography scanners. CAC scores were calculated as described by Agatston et al.^4^ Detailed methods for statistical analysis are described in the Supplementary Text.^5^

The mean (SD) age of study participants (N = 2228) was 52.4 (6.3) years, and 84.6% were men. Compared to participants with regressed NAFLD, those with persistent NAFLD were found to have a higher body mass index and a higher prevalence of diabetes and hyperlipidemia (Table). The regression of NAFLD occurred in 352 participants (15.8%). Compared to participants with persistent NAFLD, those with regressed NAFLD were more likely to be metabolically healthy. During 14,391 person-years of follow-up (median 5.3 years), 802 participants developed CAC score > 0. The fully adjusted hazard ratios for the development of CAC score > 0 in participants with regressed NAFLD compared to those with persistent NAFLD was 0.84 (95% confidence interval 0.70–0.99; Figure). In prespecified subgroups, the negative association between NAFLD regression and incident CAC (CAC score > 0) was consistent in all subgroups analyzed (all *P*-values for interaction > .10; Figure). In propensity score matching using all variables from Table, the results remained consistent (hazard ratio = 0.85, 95% confidence interval [CI] = 0.71–1.02). The annual rate of CAC progression (95% CI) in participants with regressed and persistent NAFLD at baseline was 17% (95% CI 14%–19%) and 19% (95% CI 18%–20%), respectively. The multivariable adjusted ratio of progression rates comparing participants with regressed NAFLD to those with persistent NAFLD was 0.90 (0.81, 0.99).

**Table:** Characteristics of Study Participants by Change of Nonalcoholic Fatty Liver Disease Status at Baseline (N = 2228)

Variables	Regressed (N = 352)	Persistent (N = 1876)	*P* value
Age (y)	52.4 (6.4)	52.4 (6.2)	.97
Sex			.27
Male	291 (82.7)	1594 (85.0)	
Female	61 (17.3)	282 (15.0)	
BMI (kg/m^2^)	24.2 (2.2)	25.3 (2.5)	<.01
Smoking			.75
Never	111 (31.5)	625 (33.3)	
Ever	234 (66.5)	1220 (65.0)	
Missing	7 (2.0)	31 (1.7)	
Alcohol intake			.16
None	71 (20.2)	350 (18.7)	
Light	216 (61.4)	1244 (66.3)	
Moderate	65 (18.5)	282 (15.0)	
Physical activity (per wk)			.17
Less than 3 times	166 (47.2)	978 (52.1)	
3 times or more	170 (48.3)	804 (42.9)	
Missing	16 (4.6)	94 (5.0)	
Diabetes	27 (7.7)	248 (13.2)	< .01
Hypertension	86 (24.4)	546 (29.1)	.07
Hyperlipidemia	172 (48.9)	1189 (63.4)	< .01

BMI, body mass index.

Values are mean (SD), or number (%).

**Figure:**
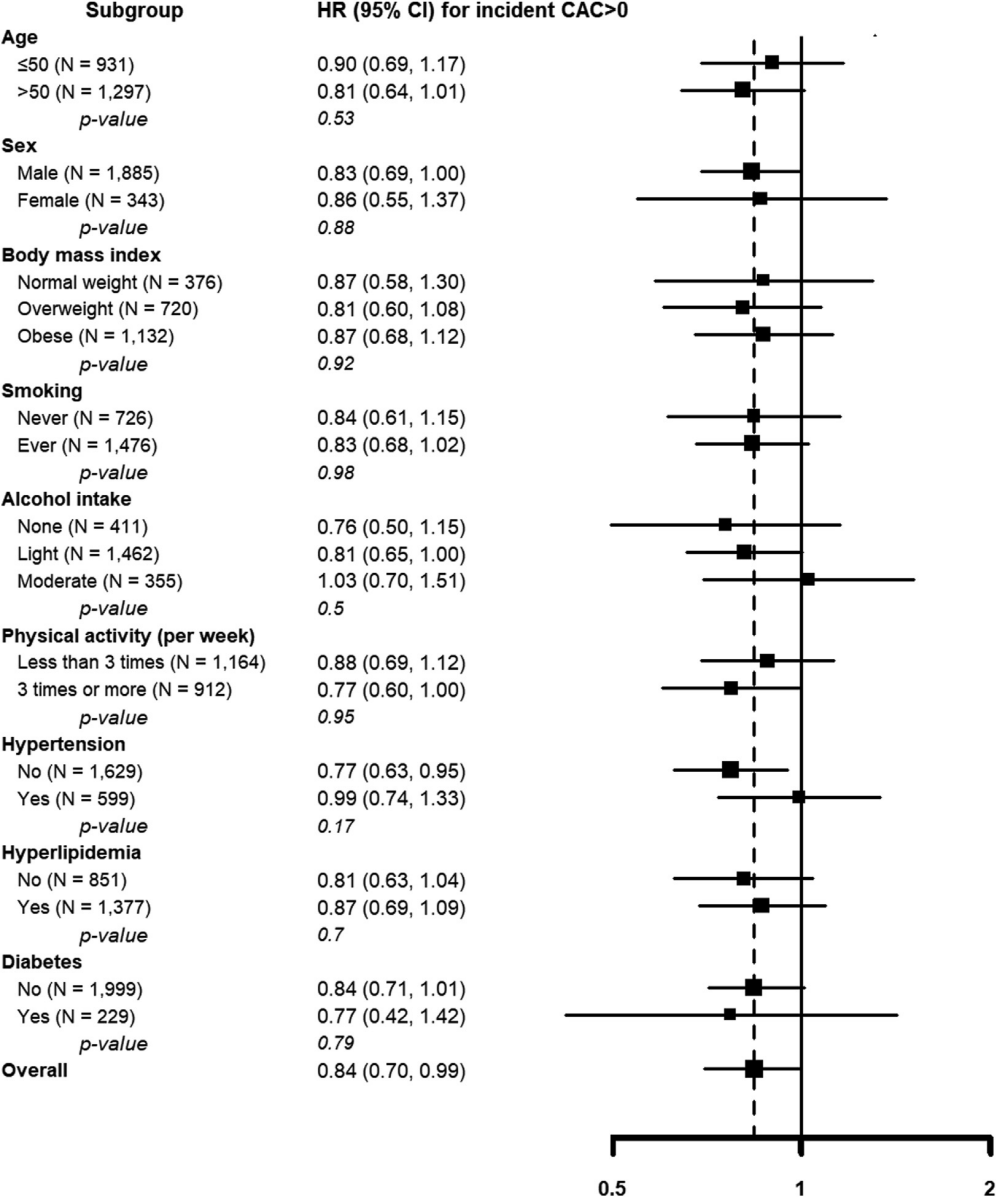
Hazard ratios for incident coronary artery calcium (CAC score > 0) comparing participants with regressed nonalcoholic fatty liver disease (NAFLD) to those with persistent NAFLD. The fully adjusted model was adjusted for sex, smoking status, alcohol intake, physical activity, hypertension, and hyperlipidemia at the second (baseline) ultrasound evaluation, and NAFLD fibrosis score at the first ultrasound evaluation (see text for details). Age, body mass index, and diabetes were not adjusted as these factors are included in the calculation of the NAFLD fibrosis score.

We previously showed that CAC progression was faster in subjects with NAFLD compared to those without NAFLD.^6^ The present study extends our findings by showing that regression of NAFLD was associated with a decreased risk of developing CAC compared to persistent NAFLD. Furthermore, the negative association between NAFLD regression and incident CAC was consistently observed regardless of age, sex, body mass index, smoking, drinking, physical activity, and comorbidities (hypertension, hyperlipidemia, and diabetes). Similarly, improvement of NAFLD has also been associated with reduced progression of carotid intima-media thickness.^7^^,^^8^ NAFLD induces a variety of changes including insulin resistance, inflammation, oxidative stress, lipid disorders, matrix metalloproteinase activity, fatty hormone levels, chronic kidney disease, and obstructive sleep apnea, which result in endothelial cell damage, inflammatory cell activation, smooth muscle cell proliferation, and atherosclerosis.^9^^,^^10^ Importantly, NAFLD is reversible, at least in earlier stages, and the reversal of hepatic fat accumulation is associated with a reduced cardiovascular risk. Limitations of our study include the use of US to identify fatty liver, which has intraobserver and interobserver variation, and the lack of information on the causes of NAFLD regression. Also, our study was based on repeated participation in health screening examinations, and participants who did not receive a second computed tomography scan for CAC scoring were excluded. However, inverse probability weighting correction for this type of selection bias did not appreciably modify the results. Similarly, including participants with only one CAC score in the mixed models also resulted in identical findings (data not shown). In spite of these limitations, our study shows that NAFLD regression is associated with a reduced risk of progression of coronary atherosclerosis and may reduce the risk of cardiovascular morbidity and mortality. Additional research is needed to establish optimal strategies for NAFLD regression and to evaluate their impact on cardiovascular risk.
