# Supplementary material for: Regression of Nonalcoholic Fatty Liver Disease Reduces the Development of Coronary Artery Calcification: A Longitudinal Cohort Study
Source: Gastro Hep Adv. 2023 Aug 16;2(8):1050–2. doi: 10.1016/j.gastha.2023.08.004 (PMC11307422; doi:10.1016/j.gastha.2023.08.004)
Supplement: Supplemental Text [file mmc1.docx]

Supplemental text

Statistical analysis

We evaluated the association between NAFLD regression and the development of any CAC score (CAC > 0) during subsequent CT scans. Participants were followed up from the visit of the second US evaluation to the detection of CAC score > 0, or to the last visit with CAC information (reference date: December 31, 2021). Since the development of CAC score > 0 occurred at an unknown time point between the visit of detection and the previous visit (interval censoring), we used a flexible parametric proportional hazards model to estimate the hazard ratio for developing CAC score > 0 comparing participants with persistent vs. regressed NAFLD at the visit of the second US (baseline).^5^ Since participants in our analyses had to have at least 2 screening visits, we used inverse probability weights (IPWs) to correct for potential selection bias. Subgroup analysis was done to evaluate whether association differs by pre-defined subgroups. In the sensitivity analysis, we calculated the propensity score using baseline characteristics and performed a 1:4 matching. We also estimated the annual change in progression of CAC scores until 12 years of follow-up (median follow up of 5.3 years) in participants who had regressed or persistent NAFLD at baseline using linear mixed models for longitudinal data with random intercepts and random slopes. In the model, we used unstructured covariance matrix with heteroskedastic error variances by CAC at index. Since CAC scores are markedly right skewed, the primary analysis used log_e_-transformed (CAC + 1) as the outcome and estimated the ratio of the annual progression rates of CAC scores [with 95% confidence intervals (CI)] comparing participants with to those without NAFLD at baseline. Statistical analyses were performed with Stata version 16.0 (StataCorp LP, College Station, Texas). All reported *P* values are 2-tailed, and comparisons with *P* < .05 were considered statistically significant.
